# Equity of health care financing in South Korea: 1990–2016

**DOI:** 10.1186/s12913-021-07308-0

**Published:** 2021-12-11

**Authors:** Tae-Jin Lee, Inuk Hwang, Hea-Lim Kim

**Affiliations:** 1 Department of Public Health Science, Graduate School of Public Health, Seoul National University, Seoul, Republic of Korea; 2 Institute of Health and Environment, Seoul National University, Seoul, Republic of Korea; 3 BK21 Center for Integrative Response to Health Disasters, Seoul National University, Seoul, Republic of Korea

**Keywords:** Kakwani index, Health care financing, Equity, Health insurance reform, Merging, Single payer

## Abstract

**Background:**

The National Health Insurance in Korea has been in operation for more than 30 years since having achieved universal health coverage in 1989 and has gone through several policy reforms. Despite its achievements, the Korean health insurance has some shortfalls, one of which concerns the fairness of paying for health care.

**Method:**

Using the population representative Household Income and Expenditure Survey data in Korea, this study examined the yearly changes in the vertical equity of paying for health care between 1990 and 2016 by the source of financing using the Kakwani index, considering health insurance and other related policy reforms in Korea during this period.

**Results:**

The study results suggest that direct tax was the most progressive mode of health care financing in all years, whereas indirect tax was proportional. The out-of-pocket payments were weakly regressive in all years. The Kakwani index for health insurance contributions was regressive but now is proportional to the ability to pay, whereas the Kakwani index for private health insurance premiums turned from progressive to weakly regressive. The Kakwani index for overall health care financing showed a weak regressivity during the study period.

**Discussion:**

The overall health care financing in Korea has transformed from a slight regressivity to proportional over time between 1990 and 2016. It is expected that these changes were closely related to the improved equity of health insurance contributions from 1998 to 2008, which was the result of a merger of the health insurance societies and an amendment in the health insurance contribution structure. These results suggest that standardizing insurance managing organizations and financing rules potentially has positive implications for the equity of healthcare financing in a country where the major method of health care financing is social health insurance.

## Background

In Korea, a universal health insurance system for its citizens and eligible foreigners has been in operation for several decades. Historically, following the introduction of a workplace health insurance in 1977, the Korean government expanded its coverage gradually. In 1988, the government implemented the health insurance scheme in rural regions and later included the self-employed in urban regions in 1989. Prior to 2000, there were three separate types of insurance schemes with more than 350 insurance societies in Korea [1]. In 2000, all organizations and finances of numerous workplace and self-employed health insurance societies were merged to create a single-payer health insurance system, which was to address disparities in the contribution schedule and to improve the equity in health care financing [1]. This system has been maintained to date.

Although the Korean health insurance is characterized as universal population coverage and a single-payer system, major issues have been raised such as controversies over the formula for calculating insurance contributions fairly and high out-of-pocket (OOP) payments [1]. For instance, workplace insurance contributions have been determined by charging a certain percentage of the wage. However, for the insurance of self-employed, contributions have been determined by estimating a household’s ability to pay based on multiple factors such as income, assets, and cars because of difficulties in adequately estimating earnings. Due to the differences in the formula for determining the contributions of workplace and self-employed health insurance subscribers, the latter tend to have a greater payment burden considering their ability to pay, especially for those in low-income groups. Additionally, self-employed health insurance subscribers in high-income groups tend to pay a smaller proportion of their ability to pay as contributions compared to workplace subscribers. This has sparked constant debates and controversies about the mechanism used to determine the contributions [2].

In addition to copayment items in the National Health Insurance, some items are not covered by insurance, for which the patient is required to pay OOP. Therefore, the proportion of OOP payments accounted for about 36% of the entire medical bill in 2018 [3]. In turn, the high OOP payment is a huge burden on the general population, especially for low-income households, leading to controversies over whether the current system is equitable to all. This also causes disparities in spending patterns between high- and low-income households in terms of the type of medical services used, including hospitalization, outpatient treatment, and medications [4].

The equity in health care financing is about whether financial contributions and/or spending on health care is made fairly based on each individual’s ability to pay. This concept focuses on achieving vertical equity, which evaluates who contributes or spends more given their ability to pay. One of the most commonly used methods to measure vertical equity in health care financing is the Kakwani index, which was originally developed to measure tax progressivity [5]. The Kakwani index was further developed to indicate whether healthcare payments on a society level are made in a financially equitable manner, and its estimation was facilitated with a convenient regression method [6]. For instance, the Kakwani index can suggest whether societal healthcare payments are made in such a manner that the rich contribute a higher proportion of their ability to pay for health care services compared to the poor (progressive) or vice versa (regressive). In addition, proportionality refers to a situation in which the proportion of income or other measures of ability to pay for health care was the same at all income levels, meaning that the rich and the poor contributed an equal proportion of their income to health care services [7]. The progressivity of a healthcare system is affected by its composition of sources for financing healthcare (i.e., direct tax, social health insurance, indirect tax, etc.) because social payments such as income tax (direct tax) are usually progressive, whereas consumption tax (indirect tax) is regressive.

Early empirical studies on the equity of health care financing in Europe and the US also used the Kakwani index for evaluation, and they suggested that while health care systems based on taxation were rather proportional or mildly progressive, those relying on social insurance or private insurance for financing tended to be more regressive. Additionally, the OOP payment in most countries was regressive [7, 8]. These generally known associations between the progressivity of health care financing and the type of healthcare systems are the results of how each society answers the question ‘who pays how much’ for health care. For instance, in a taxation-based healthcare system, the majority of health care finances are acquired from direct taxation such as income tax, which puts a higher burden on individuals with a higher income. On the other hand, in a social insurance-based health care system, the majority of funds comes from social health insurance contributions that are levied at the same rate for all individuals, therefore having different implications on the progressivity of health care financing [7].

Several additional studies were conducted in 2000s and 2010s on measuring the equity of health care financing in countries other than Western countries [9–12]. For instance, a study on the equity of health care financing among several Asian territories revealed that rich people in most low-income to mid-to-low-income countries contributed more than their ability to pay, while those in high-income countries contributed relatively less, implying that health care financing is slightly regressive [13]. This trend appeared to be related to whether a country had a universal health insurance system covering all citizens. For instance, in low-income countries that often lack a universal health insurance system, rich people pay relatively more OOP payments for health care services given their ability to pay compared to the poor because most services are not covered by the health insurance and disproportionally used by the rich. On the other hand, in high-income countries where most services are covered by a universal health insurance system, the rich contribute relatively less than the poor because health insurance contributions are usually determined as a fixed percentage of one’s ability to pay for all income levels [7].

The first study on vertical equity in health care financing in Korea was conducted in 2003 [14]. The result of computing the Kakwani index using the Korean Urban Households Survey from 1996 to 2000 showed that a direct tax was progressive, an indirect tax proportional, health insurance contributions regressive and OOP payments proportional. Although several other studies analyzed the vertical equity in health care financing in Korea, these could not consistently compare the changes in the equity of financing due to disparities in the data sources used, the years analyzed and the financing sources included [14–16].

Therefore, considering that it has been over 30 years that Korea established a universal health insurance system, it is necessary to understand how equity has changed according to the source of health care financing with a consistent methodology and to identify the main reasons for the changes in the equity of each health care financing source. The aims of this study were to analyze the yearly changes in the equity of health care financing from 1990 to 2016 using the Kakwani indices and to examine the trend in the equity of OOP payments by the type of health care services in particular. This study is expected to shed light on measures that could improve the equity in health care financing in the future.

## Methods

### Kakwani index

The Kakwani index has been used to measure the vertical equity of health care financing. It is defined as the difference (C – G) between the concentration index (C), which represents the distribution of health care financing across households in relation to the ability to pay, and the Gini coefficient (G), which represents the distribution of household expenditure (or income) indicating a household’s ability to pay. If the Kakwani index is greater than 0, it is termed “progressive,” and if the index is less than 0, it is termed “regressive.” If the index is 0 or very close to 0, it is termed “proportional.” In reality, the Kakwani index can be calculated with the convenient regression equation shown below. Here, the OLS estimate of β is equal to an estimate of the Kakwani index [17].

$$2{\sigma}_R^2\left[\frac{h_i}{{\hat{\mu}}_h}-\frac{y_i}{{\hat{\mu}}_y}\right]=\upalpha +\upbeta {R}_i+{u}_i$$

In this equation, h_*i*_ is the health care spending of household *i*; ${\hat{\upmu}}_h$ is h_*i*_ ’s average; y_*i*_ is the household expenditure of household *i*; ${\hat{\upmu}}_y$ is *y*_*i*_ ’ s average; *R*_*i*_ is the weighted fractional rank in the household expenditure distribution; and ${\sigma}_R^2$ is *R*_*i*_ ’s variance.

In this study, yearly Kakwani indices were calculated for each health care financing source including direct tax, indirect tax, health insurance contribution, OOP payment, and private insurance premium, to understand the chronological changes in the equity of each financing source. Additionally, the Kakwani index of the overall health care financing was calculated as a weighted average of the index for each financing source, using the proportion of overall health care financing accounted for by each source. Table 1 presents the mix of health care financing calculated using the Korean National Health Accounts and revenue statistics [18, 19]. The “others” category includes financing from non-profit organizations and private enterprises, which are part of the financing sources for health care but cannot be used to calculate the Kakwani index because it is not feasible to calculate the “household distribution” of such entities, and car insurance benefits that account for a very small fraction of the health care expenditure. For items in the “others” category, the ventilation method was used, assuming that these items have the same distribution as the weighted average of the Kakwani indices for the other financing sources [17].

**Table 1:** Proportion of the national healthcare payment by source of financing and year between 1990 and 2016 in Korea ^a^

	Source of financing					
Year	Direct tax	Indirect tax	Health insurance contribution	Private health insurance premium	Out-of-pocket payment	Others^b^
1990	0.033	0.041	0.314	–	0.590	0.021
1991	0.035	0.036	0.291	–	0.614	0.024
1992	0.037	0.037	0.294	–	0.609	0.023
1993	0.037	0.038	0.300	–	0.591	0.034
1994	0.039	0.035	0.301	–	0.573	0.052
1995	0.039	0.033	0.318	–	0.569	0.039
1996	0.039	0.035	0.344	–	0.542	0.040
1997	0.040	0.039	0.364	–	0.512	0.045
1998	0.053	0.038	0.399	0.014	0.452	0.045
1999	0.050	0.050	0.394	0.013	0.455	0.038
2000	0.055	0.048	0.400	0.016	0.436	0.045
2001	0.053	0.050	0.457	0.014	0.391	0.035
2002	0.053	0.054	0.445	0.015	0.401	0.032
2003	0.055	0.049	0.431	0.017	0.414	0.033
2004	0.057	0.049	0.432	0.019	0.412	0.031
2005	0.063	0.050	0.427	0.018	0.411	0.031
2006	0.066	0.050	0.436	0.019	0.399	0.030
2007	0.069	0.046	0.442	0.022	0.394	0.028
2008	0.068	0.047	0.440	0.029	0.390	0.026
2009	0.065	0.051	0.448	0.038	0.375	0.023
2010	0.062	0.048	0.454	0.041	0.373	0.022
2011	0.061	0.043	0.454	0.047	0.372	0.021
2012	0.060	0.042	0.448	0.051	0.378	0.021
2013	0.059	0.041	0.449	0.054	0.377	0.020
2014	0.060	0.041	0.447	0.059	0.371	0.022
2015	0.063	0.039	0.447	0.061	0.368	0.022
2016	0.062	0.038	0.450	0.062	0.367	0.021

^a^ calculated using KOSIS (2017) data [18, 19]

^b^ “Others” include financing from mandatory private insurance (car insurance), non-profit organization, and private companies

### Data source

This study used the Household Income and Expenditure Survey (HIES) data from 1990 to 2016 in a time series analysis on the equity of health care financing. HIES is a household-level population representative survey on income and expenditure with the longest survey history in Korea. This survey collects data on each household’s income, spending, and other relevant statuses to provide information required to measure and analyze the changes in the earning and spending of Koreans with a sample size ranging between 5500 and 7500 households between 1990 and 2016. After stratifying the country into 25 regions, the survey sample was selected using the probability proportional to size (PPS) method, with weighted values for each household. Household-level data on the ability to pay and health care financing were aggregated yearly to estimate yearly Kakwani indices.

### Variables

To measure the progressivity of health care financing, two types of data are needed: a household’s ability to pay and the household spending for each health care financing type.

#### Household’s ability to pay

In this equity analysis of health care financing, the “household expenditure” item in the HIES was used to measure each household’s ability to pay. To reflect household size, the household expenditure was adjusted using the household equivalence scale. Here, the household equivalence scale is the square root scale frequently used in recent OECD reports [20] and is shown below:

$${Y}^{\ast }=Y/{\left(A+C\right)}^{0.5}$$

where *Y*^∗^ is the equivalized household income; Y is the household income; A is the number of adults and C is the number of children in a household.

#### Spending by health care financing source

##### Direct tax

The HIES provides information on each household’s income tax, which accounts for the greatest portion of the direct tax. Taxes not available in the data, such as corporate, inheritance, and gift, were assumed to have the same distribution as income tax.

##### Indirect tax

Because the HIES does not provide information on indirect tax, the amount of indirect tax needs to be estimated by applying yearly and itemized tax rates to spending on each purchased item. Some notable indirect taxes included in this study are value-added tax (VAT), special consumption tax, and liquor tax. The special consumption tax is applied to luxurious items such as jewelry and high-quality durable items such as vehicles, and it differs for each taxable item. For most items, 10% of the VAT is applied. We estimated the amount of indirect tax by using the itemized spending data from each household. For example, because the VAT is 10% of factory price, the amount of indirect tax was calculated by multiplying the money spent by 9.09% (=10/(100 + 10)). A more detailed method of calculating the indirect tax according to the item can be found in the authors’ report [21].

##### Health insurance contribution

We used the “health insurance contribution” item, which indicates the monthly health insurance premium paid by the households, provided in the HIES.

##### Out-of-pocket payment

The OOP payment provided in the HIES includes outpatient services, inpatient services, dental and oriental medicine services, medication, health care equipment, etc. The category “medication” includes money spent on ginseng and nutritional supplements, but they were excluded from this study considering the definition provided in the OECD’s System of Health Accounts (SHA) 2011 [22]. Spending on dental services was included in outpatient service until 2002 and has been surveyed as a separate category since 2003.

##### Private health insurance

Private health insurance in Korea serves supplementary roles in health care financing because it only covers some of the OOP payments for the National Health Insurance-covered and non-covered services. To calculate the Kakwani index for private health insurance premiums, information is needed on private health insurance premiums paid by individual households. However, such information was not included in the HIES. Alternatively, this study used the information on household expenditures on private insurance premiums for two types of insurance - life insurance and fire insurance, which are non-statutory and market-based voluntary insurance schemes. The HIES surveys household expenditures for these two private insurances under two separate items. And both expenditures consisted of private insurance premiums paid for health and non-health insurances. However, it is not possible to distinguish the “health component” amount from the non-health component amount in the HIES data by itself. Therefore, we opted for the following approach to estimate the Kakwani index of private health insurances in Korea with the available data. First, we assumed that the proportion of private health insurance premiums among the expenditure for these two types of insurance was consistent across all households. Second, we computed the Kakwani indices for private insurance premiums paid for life insurance and fire insurance separately using the HIES data. Then, the weighted average of these two Kakwani indices was computed using the respective proportion of the premiums for the life and fire insurance among the total supplementary private health insurance premiums in Korea as weights. The information on the proportion of each insurance (life and fire insurance) was obtained from national level reports on insurance premiums [23, 24].

## Results

Table 2 presents the estimation results by financing source of the Kakwani indices in Korea between 1960 and 2016. Direct tax was the most progressive item among all financing sources in all years while progressivity increased from 1994 and onward peaking in 2008 with a Kakwani index value of 0.373. Then, the progressivity decreased gradually with a value of 0.330 in 2016. Financing through indirect tax showed positive values until 1997, after which the Kakwani indices turned negative. However, the absolute value was still close to 0 indicating that indirect tax was proportional to the ability to pay.

**Table 2:** Kakwani Indices by source of healthcare financing and year between 1990 and 2016 in Korea

	Sources of financing					
Year	Direct tax	Indirect tax	Health insurance contribution	Private health insurance premium ^a,b^	Out-of-pocket payment	Overall
1990	0.268	0.040	−0.107	–	− 0.040	− 0.048
1991	0.276	0.041	−0.125	–	− 0.052	− 0.059
1992	0.242	0.036	−0.135	–	− 0.039	− 0.055
1993	0.232	0.042	−0.129	–	− 0.060	− 0.066
1994	0.200	0.027	− 0.124	–	−0.063	−0.068
1995	0.246	0.024	−0.113	–	−0.054	−0.058
1996	0.242	0.025	−0.127	–	−0.069	−0.074
1997	0.254	0.018	−0.144	–	−0.063	−0.077
1998	0.320	−0.010	−0.141	0.153	−0.073	−0.074
1999	0.333	−0.003	−0.106	0.099	−0.061	−0.054
2000	0.347	0.004	−0.078	0.064	−0.069	−0.043
2001	0.334	−0.004	−0.072	0.224	−0.068	−0.040
2002	0.329	−0.002	−0.054	0.131	−0.067	−0.033
2003	0.340	−0.015	−0.030	0.001	−0.071	−0.025
2004	0.360	−0.025	−0.013	0.023	−0.059	−0.011
2005	0.350	−0.023	−0.019	−0.004	−0.061	−0.013
2006	0.360	−0.028	0.003	0.030	−0.086	−0.010
2007	0.365	−0.027	0.017	0.024	−0.093	−0.005
2008	0.373	−0.033	0.027	0.046	−0.113	−0.007
2009	0.352	−0.029	0.004	−0.014	−0.098	−0.015
2010	0.359	−0.038	−0.002	0.011	−0.086	−0.013
2011	0.361	−0.024	0.009	0.022	−0.106	−0.014
2012	0.345	−0.025	0.012	−0.070	−0.103	−0.018
2013	0.340	−0.021	0.008	−0.081	−0.104	−0.021
2014	0.320	−0.017	0.012	−0.069	−0.116	−0.024
2015	0.311	−0.024	0.016	−0.056	−0.111	−0.018
2016	0.330	−0.030	0.023	−0.050	−0.111	−0.014

^a^ As the ratio of supplementary private health insurance among health care financing sources is available since 1998, the Kakwani index of private insurance was calculated from 1998

^b^ Considering that life insurance companies began selling supplementary private health insurance since September 2005, the Kakwani index of private health insurance between 1998 and 2005 was calculated using only the fire insurance; since 2006, the Kakwani index was calculated by obtaining the weighted average of life insurance and fire insurance

The Kakwani index for health insurance contribution was − 0.107 in 1990, which was slightly regressive, and there was no notable change until the regressivity started to decrease in 1998. In 2016, it was almost proportional with a value of 0.023. The Kakwani index for OOP payment showed negative values throughout the period, indicating regressivity, although it was close to 0. However, a slightly increasing trend was noted in the regressivity since 2008. The Kakwani index for private health insurance showed progressivity from a value of 0.153 in 1998 to 0.224 in 2001. However, the progressivity started gradually decreasing in 2003, showing a slight regressivity since 2012.

**Fig. 1:**
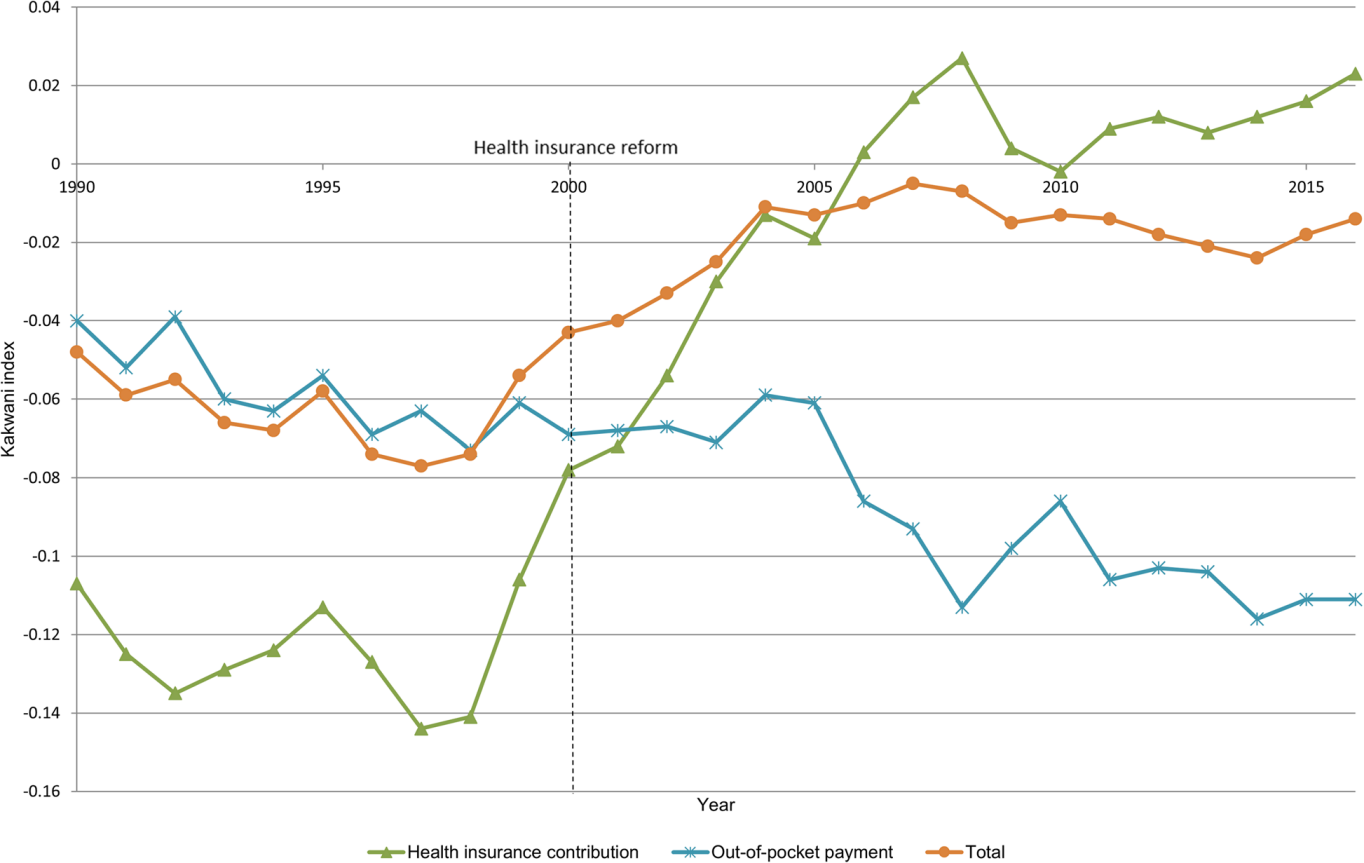
Kakwani indices for the two most important health care financing sources – health insurance contribution and OOP payment – in addition to total health care expenditure between 1990 and 2016 in Korea

The Kakwani index for overall health care financing showed a small negative value, i.e., weak regressivity, throughout the years. A detailed analysis indicated that the Kakwani index for overall health care financing was − 0.048 in 1990 and − 0.077 in 1997, showing a slightly increased regressivity. The regressivity decreased in the following years, showing a Kakwani index of − 0.014 in 2016, which is almost proportional. Figure 1, which was produced using data from Table 2, shows the Kakwani indices of the health insurance contribution and OOP payment, which together comprised over 80% of the overall health care financing. As shown in the figure, the Kakwani indices of the two major financing sources changed in the opposite direction over the years.

**Fig. 2:**
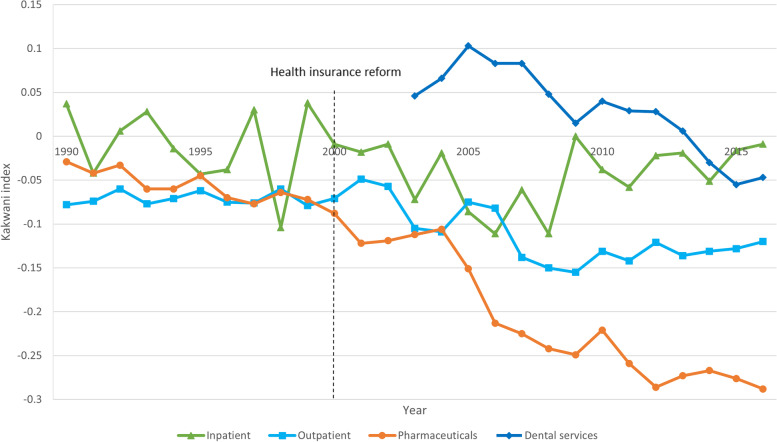
Kakwani indices of OOP payment by type of service between 1990 and 2016 in Korea

The Kakwani indices for each year according to the type of health care service are shown in Fig. 2. The OOP payment for inpatient treatment is either almost proportional or regressive to the ability to pay for all years. The OOP payments for outpatient treatment and medication are regressive compared to the ability to pay for all years, and the regressivity showed an increasing trend towards the end of the period. In particular, the regressivity for medication was much greater in the 2000s than it was in the 1990s, and it was the most regressive of all four service types. The Kakwani index for the dental OOP payment indicated a decreasing progressivity since 2005, showing negative values again from 2014, contributing to the increase of the regressivity for the overall OOP payments.

## Discussion

To summarize the results of this study, the direct tax was progressive; the indirect tax was proportional; the OOP payment was regressive, and the health insurance contribution was regressive and then became proportional to the ability to pay. Detailed elaborations on each source of financing are provided below.

The finding that direct tax is the most progressive is generally shared by previous studies [7, 8, 14]. From a longitudinal perspective, the progressivity of the direct tax improved to the greatest extent between 1994 and 2000 and then gradually improved since 2000 This is most likely related to the government enacting many income tax-related policies focusing on the redistribution of income to resolve income inequality caused by rapid economic growth. To elaborate, the following policies might have affected the change. Between 1994 and 2000, major income tax-related policies such as the real-name financial system (1993), real-name property ownership system (1995), and comprehensive taxation on financial income (1996) were implemented. Among them, the first two made financial and property transactions among the high-income population more transparent while the last ensured that more income tax was imposed on households with revenue from high-interest products or dividends [25]. Additionally, the amendments on the deduction for wages and income based on wage levels and the introduction of the maximum tax bracket to the high-income group, which were major changes after 2000, led to a relatively greater income tax burden on wage workers who belonged to the tax group exceeding the marginal tax rate of 24% [26].

Although indirect tax is normally considered regressive to the ability to pay [6, 7], this study found that it was almost proportional during the study period. This result is identical to a previous study conducted in Korea [13]. However, close analysis revealed that the sign of the Kakwani index changed from (+) to (−). Changes in the progressivity of the indirect tax may have been partially caused by changes in taxes, which could be affected by income level, such as a special consumption tax and tobacco tax. In other words, the progressivity of the indirect tax worsened as items subject to the special consumption tax, which burdened high-income individuals, gradually disappeared. In addition, tobacco that had a special consumption tax imposed on it since 2015 is expected to have contributed to the worsening of the progressivity because tobacco is generally known to be consumed relatively more by low-income groups than by high-income groups [27].

The Kakwani index for the health insurance contribution showed that the regressivity decreased over time. The equity of the health insurance contribution improved to the greatest extent between 1998 and 2008. Several major changes could have affected the equity of the health insurance contribution during this era, and the biggest change would be the merger of the health insurance societies and finances in 1998–2001. Since the introduction of workplace health insurance in Korea in 1977, health coverage was extended by introducing regional health insurance for the self-employed in rural areas in 1988 and in urban areas in 1989, respectively. In this process, there existed hundreds of health insurance societies with different budgets and finances, and such disparity was the reason for the differences in contributions. In other words, rich societies were able to impose a low contribution rate while poor societies were only able to impose a high contribution rate, causing regressivity in the health insurance contribution. Under these circumstances, a stepwise change to a single-payer system - the merger of the regional health insurance societies in October 1998, followed by the merger of workplace insurance societies in July 2000 - contributed significantly to the improvement of equity in the health insurance contribution. Specifically, it was made possible to set a nationally uniform contribution rate in the single-payer system generated from the merging health insurance societies [28]. Due to this change, the Kakwani index of the health insurance contribution greatly improved from − 0.144 in 1997 to − 0.072 in 2001. Next, the equity of the health insurance contribution was improved to a good extent in 2007–2008, and this appears to have been caused by changes in the method for calculating the health insurance contributions. In 2007, the government abolished the ranking system for calculating contributions and began calculating insurance contributions based on actual wages (for workplace insurance holders) and insurance scores based on assets, etc. (for regional insurance holders). To illustrate this, the health insurance contribution of a workplace insurance holder was levied based on their wage according to a “standard wage ranking” chart until 2006. However, since 2007, the contribution amount was calculated by multiplying the actual monthly wage by a contribution rate, enabling the government to impose a health insurance contribution that is more proportional to wage. These results suggest that unifying or standardizing insurance managing organizations and financing rules potentially has positive implications for the equity of health care financing in a country where the major method of health care financing is social health insurance, and the social health insurance is managed sparsely or operated under different standards for different sub-populations. Indeed, in several other countries where the health insurance funds (HIFs) were merged to create a single-payer health insurance system or reduce the number of HIFs, the equity in health care financing has been reported to be improved as well. Specifically, the share of the OOP health care payment, which is usually the most regressive form of health care financing, was reduced to 19% in Turkey and 12% in Thailand several years after the merger of their HIFs, from nearly 30% before the merger [15].

The Kakwani index of the OOP payment was close to proportional in 1990, but the regressivity gradually and consistently increased over time. Although the degree is different, other studies also showed that the OOP payment after the 1990s was regressive [16, 29]. Normally, the OOP payment tends to be paid regardless of the income level, thus increasing the possibility of regressivity. However, the continual increase of the regressivity was rather notable. In particular, the slope of the increase in the regressivity was greater after 2006, and this is partially related to the limited health insurance coverage, which stalled around 63% during this period [3]. In other words, when health care expenditure continues to increase but the coverage rate by the insurance stalls, the actual spending of each household increases. As such, the regressivity might have increased as people with a low income ended up paying more than their ability to pay.

The Kakwani index for private health insurance premium was seen to become more regressive. This is related to the fact that around 70% of the Korean households have private health insurance [30]. In other words, to take care of an OOP health payment that is around 37% of the total health care expenditure, more than two-thirds of the households bought private insurance. And the proportion of low-income households among the private insurance subscribers was significant, contributing to the greater regressivity of the private insurance premium.

The Kakwani index for overall financing showed a slightly increasing trend in the regressivity between 1990 and 1997, followed by a downward trend, resulting in a value of − 0.014 in 2016, which was almost proportional. This is related to the time-based changes in the two greatest sources of health care financing, i.e., health insurance contributions and OOP payments. In other words, (i) while the regressivity of the health insurance contribution decreased, its ratio increased among the overall financing, and (ii) the ratio of the OOP payment in overall financing sources decreased while its regressivity increased.

Finally, the trend for the OOP payment based on the type of health care services showed that the Kakwani index for inpatient services was proportional to the ability to pay, and the indices for outpatient services and medication showed a continuous increase in the regressivity. Considering the trend that low-income families have higher possibilities of having health issues, the result reflects that the low-income population may have unmet demands for inpatient services and instead use the outpatient services or medication disproportionately more because they are financially less burdensome.

This study has several limitations. First, because the HIES does not provide information for each household’s expenditure on supplementary private health insurance, the study had to assume that the proportion of supplementary private health insurance premium among the premiums paid by each household for life insurance and fire insurance was consistent. Therefore, in interpreting the Kakwani index for private insurance, this assumption must be considered. Especially, given the recent increase in the enrollment of supplementary private health insurance in Korea, which puts a relatively higher burden on low-income groups, it is possible that the regressivity of the Kakwani index for private health insurance is more pronounced than what was computed in this study. Second, obtaining the equity of the overall health care financing requires the ratio of each financing source. However, the data on private health insurance is available only after 1998 and was not included in calculating the Kakwani index of the overall health care financing before 1998. However, since the financing by private insurance was less than 2% of the overall health care financing before supplementary private health insurance became available, this would not have made a meaningful difference. Third, the OOP spending on dental services prior to 2002 was considered outpatient spending. Therefore, the Kakwani index for dental services prior to 2002 was not obtained separately, and thus, caution must be exercised when interpreting the data. Fourth, it is possible that social changes that are not directly in the realm of health care financing may have affected the vertical equity of health care financing during the study period. However, to the best of the authors’ knowledge, there were no other systematical/tangible changes of great significance, like the mergers of social insurances, to affect the progressivity of health care financing in Korea. In addition, it is extremely difficult to distinguish, if not impossible, social events that have indirectly affected health care financing through long and complicated chains of events. However, key changes in the social health insurance scheme, which accounts for the majority of the overall health care financing in Korea, were controlled for, which was in line with the objective of this study. Lastly, this study was not able to include more recent data in the analysis because the sample and survey methods for the HIES in Korea have been modified since 2017, making comparisons with previous years not feasible.

## Conclusions

This study observed and analyzed the changes in the equity of health care financing in Korea for nearly a 30-year period. The overall health care financing transformed from slight regressivity to proportional over time. Such a change is closely related to the improvement in the equity of the health insurance contributions from 1998 to 2008, which was mainly due to the mergers of health insurance societies and the amendment in the health insurance contribution structure. Since July 2018, health insurance contributions in Korea have been restructured to reflect more of an individual’s actual ability to pay. Therefore, additional assessment is necessary for whether the equity in health insurance contributions and overall health care financing has improved.

## Data Availability

The data that support the findings of this study are available from Statistics Korea but restrictions apply to the availability of these data, which were used under license for the current study, and so are not publicly available. Data are however available from the authors upon reasonable request and with permission of Statistics Korea.

## Abbreviations

OOP: Out-of-pocket
HIES: Household Income and Expenditure Survey
PPS: Probability proportional to size
VAT: Value-added tax
SHA: System of Health Accounts
HIF: Health insurance fund
